# High-risk spatiotemporal patterns of cutaneous leishmaniasis: a nationwide study in Iran from 2011 to 2020

**DOI:** 10.1186/s40249-023-01103-1

**Published:** 2023-05-15

**Authors:** Neda Firouraghi, Robert Bergquist, Munazza Fatima, Alireza Mohammadi, Davidson H. Hamer, Mohammad Reza Shirzadi, Behzad Kiani

**Affiliations:** 1 Department of Medical Informatics, School of Medicine, Mashhad University of Medical Sciences, Mashhad, Iran; 2 Ingerod, Brastad, Sweden; 3 Formerly with the UNICEF/UNDP/World Bank/WHO Special Program for Research and Training in Tropical Diseases, World Health Organization, Geneva, Switzerland; 4 Department of Geography, The Islamia University of Bahawalpur, Bahawalpur, Punjab Pakistan; 5 Department of Geography and Urban Planning, Faculty of Social Sciences, University of Mohaghegh Ardabili, Ardabil, Iran; 6 Department of Global Health, Boston University School of Public Health, Boston, MA USA; 7 Section of Infectious Diseases, Department of Medicine, Boston University School of Medicine, Boston, MA USA; 8 Center for Disease Control and Prevention (CDC), Iran Ministry of Health & Medical Education, Tehran, Iran; 9 Centre de Recherche en Santé Publique, Université de Montréal, 7101, Avenue du Parc, Montréal, Canada

**Keywords:** Cutaneous leishmaniasis, Spatial epidemiology, Geographical Information Systems, Spatiotemporal analysis, SaTScan, Spatial scan statistics, Neglected tropical diseases, Spatiotemporal clustering, Iran

## Abstract

**Background:**

Cutaneous leishmaniasis (CL) is a wide-reaching infection of major public health concern. Iran is one of the six most endemic countries in the world. This study aims to provide a spatiotemporal visualization of CL cases in Iran at the county level from 2011 to 2020, detecting high-risk zones, while also noting the movement of high-risk clusters.

**Methods:**

On the basis of clinical observations and parasitological tests, data of 154,378 diagnosed patients were obtained from the Iran Ministry of Health and Medical Education. Utilizing spatial scan statistics, we investigated the disease’s purely temporal, purely spatial, spatial variation in temporal trends and spatiotemporal patterns. At *P* = 0.05 level, the null hypothesis was rejected in every instance.

**Results:**

In general, the number of new CL cases decreased over the course of the 9-year research period. From 2011 to 2020, a regular seasonal pattern, with peaks in the fall and troughs in the spring, was found. The period of September–February of 2014–2015 was found to hold the highest risk in terms of CL incidence rate in the whole country [relative risk (*RR*) = 2.24, *P* < 0.001)]. In terms of location, six significant high-risk CL clusters covering 40.6% of the total area of the country were observed, with the *RR* ranging from 1.87 to 9.69. In addition, spatial variation in the temporal trend analysis found 11 clusters as potential high-risk areas that highlighted certain regions with an increasing tendency. Finally, five space-time clusters were found. The geographical displacement and spread of the disease followed a moving pattern over the 9-year study period affecting many regions of the country.

**Conclusions:**

Our study has revealed significant regional, temporal, and spatiotemporal patterns of CL distribution in Iran. Over the years, there have been multiple shifts in spatiotemporal clusters, encompassing many different parts of the country from 2011 to 2020. The results reveal the formation of clusters across counties that cover certain parts of provinces, indicating the importance of conducting spatiotemporal analyses at the county level for studies that encompass entire countries. Such analyses, at a finer geographical scale, such as county level, might provide more precise results than analyses at the scale of the province.

**Graphical Abstract:**

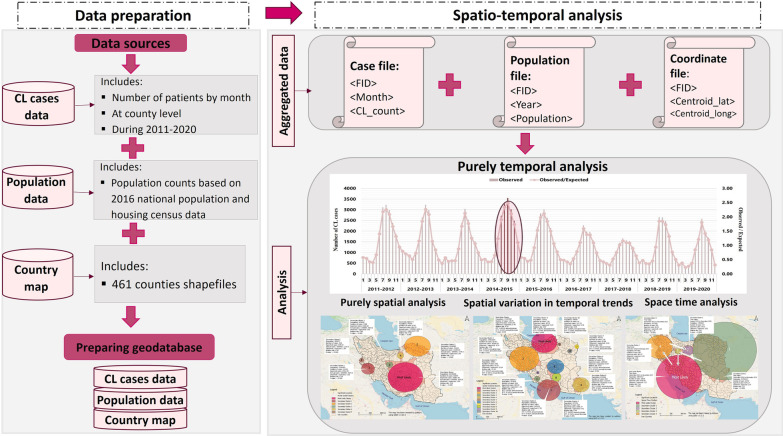

**Supplementary Information:**

The online version contains supplementary material available at 10.1186/s40249-023-01103-1.

## Background

Cutaneous leishmaniasis (CL) is a multi-reservoir disease transmitted by female sand flies (Phlebotominae) [1–3]. It has two major types: a zoonotic form where animals are the reservoir and an anthroponotic one where humans spread the infection [4]. CL might cause permanent facial scars and is considered an emerging, neglected infectious disease. No effective drugs or vaccines are available, but medical interventions can be used to manage CL ulcers [1]. CL transmission depends on environmental changes that favour the habitat of vector species close to human settlements [5]. Sand flies cause the wide geographical spread of the infection, especially in tropical and subtropical areas [6, 7]. Between 600,000 and 1 million new cases of CL occur annually [3], and it is endemic in various regions including the Americas, Eastern Mediterranean, North Africa, and parts of Central Asia [3, 8].

Infectious diseases vary geographically and over time [9]. Spatiotemporal analysis is useful for identifying high-risk CL areas [10, 11], dynamic patterns, disease outbreaks, and populations at risk [12, 13]. The geographical distribution of CL is driven by complex interactions between parasite reservoirs, parasites, vectors, climate, ecology, political instability, poverty, and socioeconomic changes [14–16]. All of these drivers could potentially cause an uneven distribution of the disease that can be explored by spatiotemporal analysis [17]. For instance, a study in the Republic of Colombia identified high-burden clusters of CL associated with deforestation, livestock, and socioeconomic factors [18]. Other studies in Syria and Jordan [19], and Brazil [20] extracted spatiotemporal variations of CL cases, allowing for the classification of high-priority areas.

CL is the most prevalent protozoal disease in Iran [21], which has one of the world’s highest annual incidence rates and is surrounded by many other CL-endemic countries [3]. Non-immune populations cross borders, leading to the spread of the infection to larger areas [14]. CL is a mandatory notifiable disease in Iran that is required to be reported to the department of preventive diseases [22, 23], and an estimated 20,000 cases are reported annually [24], with geographical variation in prevalence [25]. Several spatial analysis techniques have been employed in Iran to study CL and its associated factors. For instance, studies have used spatiotemporal clustering methods to examine space-time patterns and spatial distribution of CL [11, 26, 27]. In addition, researchers have used regression analysis to investigate the spatial relationship between CL incidence and various climate and environmental factors [28]. A recent study in an endemic urban area of Mashhad as the second most populated city in Iran employed various spatial and temporal analysis techniques, including empirical Bayesian smoothing and spatial autocorrelation analysis, to identify clusters and outliers across census tracts, and spatial scan statistics to explore the spatiotemporal trend of CL in the study area [10].

Despite the high endemicity of CL, there is currently no comprehensive spatiotemporal analysis of CL at the county level in Iran. Ghatee et al. [14] advised applying geospatial research in Iran so that regions can be stratified based on the probability of CL and with the aid of such hazard maps. Spatial analysis at the county level might provide more detailed and localized information about the distribution of a disease, allowing for more precise identification of areas with high disease burden and insights into local risk factors and interventions. This can help to identify areas that may have been overlooked in previous studies that analyzed data at the provincial level, leading to a more comprehensive understanding of the spatial distribution of the disease and enabling the development of targeted prevention and control strategies [29, 30]. Additionally, conducting analysis at the county level might improve resource allocation and enhance the accuracy of disease mapping [31]. Therefore, this study aims to fill this gap by conducting a nationwide spatiotemporal analysis of CL at the county level for the entire country with the most updated data. The aforementioned information can be utilized by policymakers to make informed decisions and formulate customized interventions that are effective in managing the control and prevention of CL.

## Methods

### Study area

A mountainous nation, Iran is the fourth-largest country in Western Asia covering 1,648,195 km^2^. The country is bordered by Azerbaijan, Armenia, and Turkmenistan in the north; by Pakistan and Afghanistan in the east; and by Turkey and Iraq in the west. The population is approximately 85 million. Iran consists of 31 provinces and 461 counties in total [32]. Additional file 1 provides insights into the spatial distribution of precipitation and temperature patterns, highlighting areas with high or low levels of rainfall and temperature variation.

### Data sources

In this study, three distinct sources of data were aggregated at the county level. To conduct spatiotemporal analysis, the data was structured in the SaTScan input data format, which comprises three files. These data have been publicly made available through Additional files 2, 3, 4, and 5.

1. Data on CL instances were acquired from the Iran Ministry of Health and Medical Education. This information contained the number of patients per county, by month and year over the period March 2011 to March 2020. The data were collected at the county level using the official Persian calendar, whose year begins on March 20 (Gregorian calendar). The two most common species of CL in Iran are *Leishmania tropica* and *Leishmania major* that are not separated in this study. In this study, all CL cases were diagnosed and confirmed by microscopic examination of smears taken from the margin of lesions. As CL is a mandatory notifiable disease in Iran, every diagnosed case of CL is required to be reported to the Ministry of Health [23]. Therefore, we were able to capture all patients with CL visiting any healthcare centers in Iran during the study period.
2. Population data were derived from the 2016 National Population and Housing Census conducted by the Statistical Center of Iran [33].
3. The Ministry of the Interior provided the vector map of counties as shapefiles, based on the most recent geopolitical subdivision, which amounts to 461 counties.

### Spatiotemporal analysis

A retrospective spatiotemporal scan statistical analysis was conducted using Kulldorff’s methodology [10, 13] to identify high-risk clusters in a purely temporal, purely spatial, or spatiotemporal context. The analysis utilized a pre-defined scanning window that traversed space and time, and the number of observations within the window was compared to that outside the window. The relative risk (*RR*) was calculated by dividing the estimated risk within the cluster by that outside the cluster under the null hypothesis of equal disease risk within and outside the cluster. The estimated risk was calculated by dividing the number of observed cases by that of expected cases. The log likelihood ratio (*LLR*) was used to rank the clusters, and the cluster with the highest *LLR* was identified as the most likely cluster, while others were classified as secondary. Secondary clusters that have no common geographic area with the most likely cluster may be of great interest as they are able to reject the null hypothesis on their own strength and are always reported. Overlapping clusters were not considered in this study. The practical difference between the most likely and secondary clusters identified by spatial scan statistics is that the most likely clusters have the strongest evidence of disease clustering, while secondary clusters may also exhibit evidence of disease clustering, but not as strong as the most likely clusters. These clusters, with significantly large likelihood ratio are calculated without considering the most likely cluster. Secondary clusters that are geographically distinct from the most likely cluster provide additional evidence that the disease is not randomly distributed. Both types of clusters provide important information about disease distribution in the study area, guiding public health interventions and resource allocation [34, 35]. The *P*-value for discovered clusters was obtained using Monte Carlo simulation, with 999 simulations predetermined to increase the test’s reliability [36]. The null hypothesis was rejected if the maximum *LLR* of the real dataset fell within the top 5% of the corresponding clusters in the random dataset, indicating significance at the 0.05 level. To prevent defining unrealistic clusters, the window size was set to be between 0 and 25% of the population at risk, as excessively large cluster sizes may be difficult to interpret. A larger window size may increase sensitivity, but also increase the risk of false positives. A smaller window size may increase specificity, but may miss smaller clusters. A 25% window size was considered a reasonable compromise to detect disease clusters that were not too small or too large [37].

In our study, we utilized Poisson distribution to model the occurrence of CL as it met several criteria [38–40]. Firstly, CL occurrence is a countable data in the time and space interval, which is suitable for modelling using the Poisson distribution [12, 39, 40]. Secondly, the probability of a new CL occurrence in any time and space level is very low, making it a rare event that can be modelled using the Poisson distribution. Additionally, CL transmission occurs randomly and independently through the bite of infected female phlebotomine sand flies, and person-to-person transmission is rare [41, 42], making the occurrence of CL independent at the individual level. Finally, only one CL occurrence can happen at a given time or space, means a person can either be affected or not affected by CL at any given time [12, 43].

We used ArcGIS, v. 10.6 (ESRI, Redlands, CA, USA) [44] for data preparation. The SaTScan™ software, v 9.6.1 (Martin Kulldorff, Harvard Medical School, Boston and Information Management Services, Inc.) [45] was applied for spatial analysis. Also, QGIS v.3.22.3 (free and open-source Geographic Information Systems, the Federal Department of Town and Country Planning, Peninsular Malaysia, USA) [46] was used for mapping and visualization.

#### Purely temporal analysis

A purely temporal analysis was employed to identify clusters of high-risk significance that occur only in the temporal dimension (time), without considering spatial patterns. This is accomplished by using a scanning window that scans only in the temporal dimension to identify the number of occurrences during the study period, such as monthly peaks. The length of time aggregation was set to 1 month.

#### Purely spatial analysis

A circular scanning window was applied to the entire map of the country, generating an endless number of spatial windows as potential clusters. A candidate cluster was defined as a grouping of counties centered on the coordinate points within the scanning window [36].

#### Spatial variation in temporal trends

The significant clusters that were detected indicated a difference in temporal trends between the interior and exterior of the scanning window. The null hypothesis stated that the temporal trends inside and outside the window were the same [36]. Temporal trends were calculated within the scanning windows as internal time trend (*ITT*) and outside the windows as external time trend (*ETT*). *ITT* represented the variation in temporal trend for CL occurrence within a cluster, and *ETT* referred to the temporal trend of CL occurrence in all other regions. Therefore, the analysis evaluated the statistical significance of temporal trends rather than the overall disease rate being higher or lower. This discrepancy could be attributed to either a higher incidence rate of CL in the clustered area at the end of the period or a lower incidence rate in the detected region at the beginning of the period.

#### Space-time analysis

**Fig. 1:**
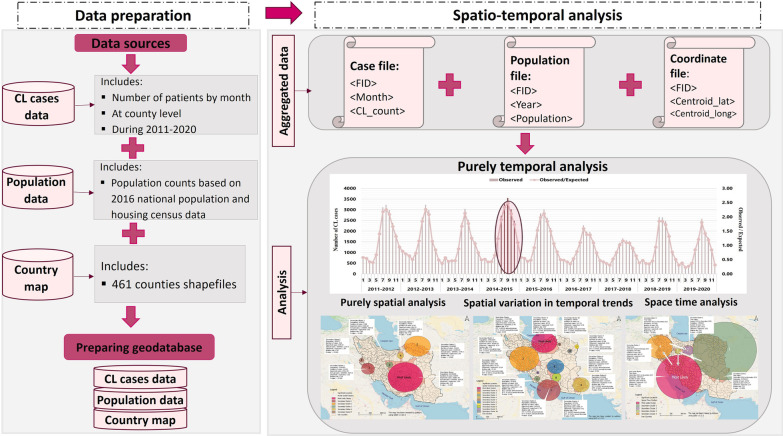
Methodological framework to identify cutaneous leishmaniasis temporal, spatial, and spatiotemporal patterns

A cylindrical scanning window with a circular base was employed to examine spatial patterns and a height for measuring the time period of clusters. This window traverses all feasible regions and periods. The goal of this method is to determine if there is any cluster pattern in terms of the spatial and temporal distribution of the illness [36, 47]. Figure 1 illustrates the entire research process as a framework for the methodology. Additional file 1 shows the name of Iranian provinces and their location in the country.

## Results

### Descriptive results

**Fig. 2:**
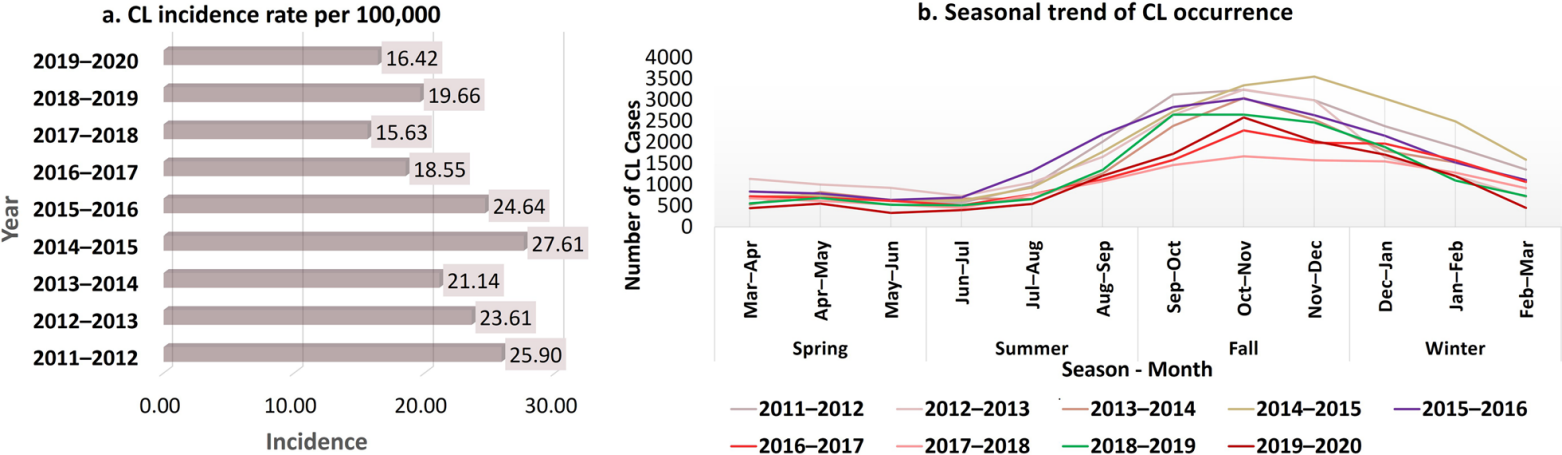
Temporal trend of cutaneous leishmaniasis cases, reported in Iran from March 2011 to March 2020

Between March 2011 and March 2020, there were 154,378 confirmed cases of CL in Iran. The incidence rate of CL shows an overall decline in disease occurrence, although there were some years with an increase in cases (Table 1, Fig. 2a). A seasonal pattern is also observed, as the number of CL cases peaked in the fall and declined in the spring throughout the study period (Fig. 2b). Specifically, CL cases were most frequent in October and November, indicating an increase in CL incidence after late summer. This trend continued throughout the winter months with a gradual decline in CL cases.

**Table 1:** Number of new identified cutaneous leishmaniasis cases by season during 2011–2020 in Iran

Year	Spring	Summer	Autumn	Winter	Total
2019–2020	1313	2138	6328	3345	13,124
2018–2019	1752	2498	7759	3704	15,713
2017–2018	1801	2270	4688	3730	12,489
2016–2017	2020	2386	5827	4590	14,823
2015–2016	2254	4187	8482	4770	19,693
2014–2015	2054	3312	9610	7092	22,068
2013–2014	1976	2579	7953	4386	16,894
2012–2013	3046	3416	8863	3546	18,871
2011–2012	2217	3537	9337	5612	20,703
Total	18,433	26,323	68,847	40,775	15,4378

### Purely temporal clusters

**Fig. 3:**
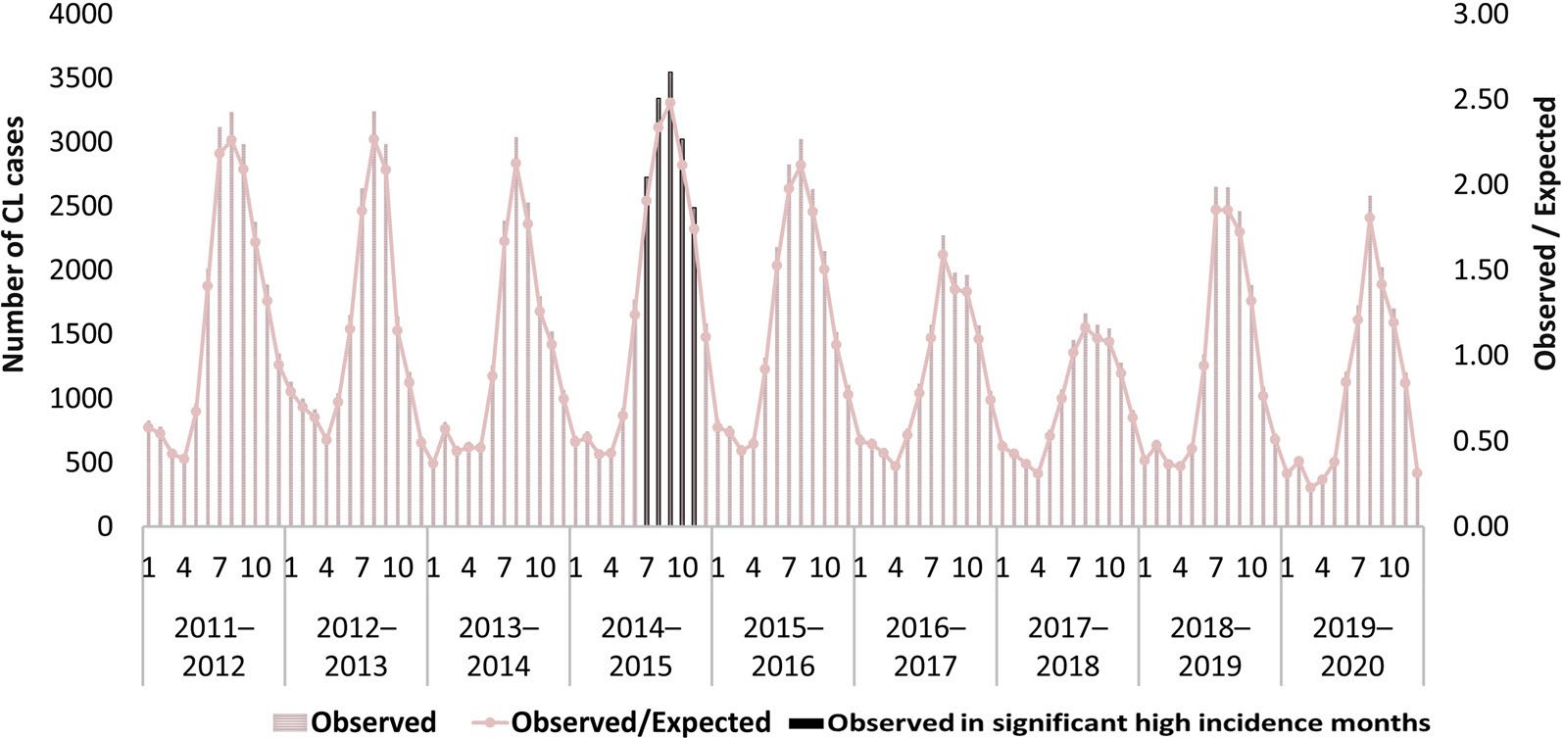
Purely temporal scanning of cutaneous leishmaniasis cases, reported in Iran during March 2011 and March 2020

The purely temporal scanning for high-rate CL clusters revealed a downward trend in the number of CL cases from 2011 to 2014; however, this trend reversed in September–February of 2014–2015, when the rate reached its peak. This time frame was identified as the only significant cluster (*RR* = 2.24, *P* = 0.001). During this time frame, 15,120 instances of CL were recorded (Fig. 3). The subsequent decrease then began and lasted until the end of 2017–2018. CL number has not followed the declining trend observed in recent years during the last 2 years.

### Purely spatial clusters

**Fig. 4:**
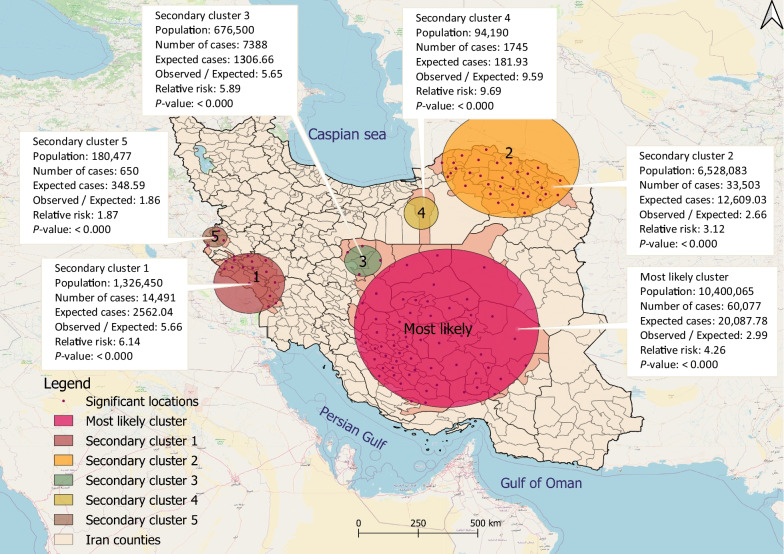
High-risk purely spatial clusters of cutaneous leishmaniasis cases in Iran between March 2011 and March 2020

Geographical scanning identified six significant high-risk clusters of CL with a radius of 852.87 km, covering 40.6% of the total area of the country (*P* < 0.001). These clusters included 24.0% of the total population (Fig. 4). The *RR* for these clusters ranged from 1.87 to 9.69 and included 118 counties in 16 provinces. Approximately three-quarters (76.4%; 117,985 cases) of the total number of CL cases were found within these spatial clusters. The cluster with the highest *LLR*, considered the most likely cluster (*LLR*: 32,478.79; *P* ≤ 0.05), was scattered from the south-central to the central regions of Iran, covering 58 counties in Isfahan, Fars, Hormozgan, Kerman, South Khorasan, and Yazd provinces. Secondary high-risk clusters were also identified in the southwestern, northeastern, central, and western regions of Iran.

### Spatial variation in temporal trends

**Fig. 5:**
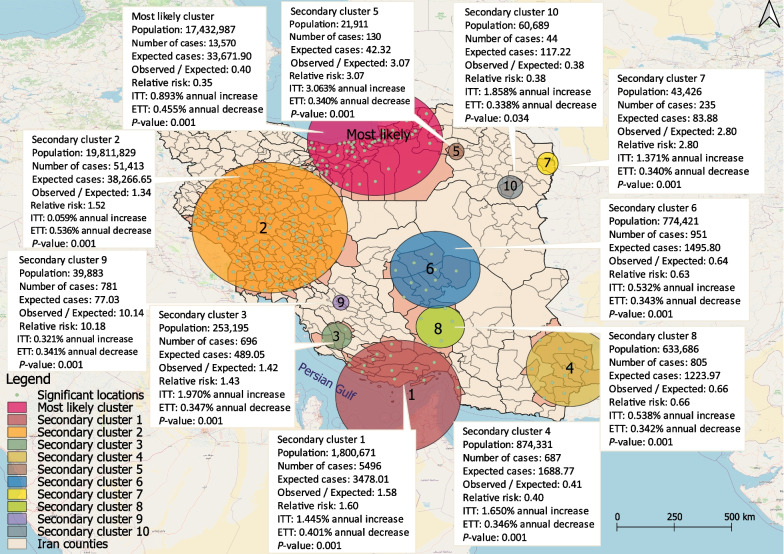
Spatial variation in the temporal trend of cutaneous leishmaniasis cases, reported in Iran between March 2011 and March 2020. *ITT* internal time trend; *ETT* external time trend

Geographic variation in the time trend analysis from 2011 to 2020 detected eleven significant CL clusters (*P* < 0.05), including one most likely cluster and ten secondary clusters. These clusters cover a total radius of 1325.2 km, accounting for 56.6% of the country’s total area and nearly half of all CL cases [48.5% (74,808)] across 218 counties (Fig. 5). *ITT* increased annually across all clusters, while *ETT* decreased annually. The most likely cluster, with a *LLR* of 1069.9, comprised 52 counties in the northern and central-northern regions of Iran, including Tehran, Alborz, Semnan, Mazandaran, North Khorasan, and Golestan provinces. Secondary clusters were located primarily in the western, southern, south-eastern, central-southern, and central-western regions of Iran. The highest annual growth in CL cases was observed in secondary cluster no. 5 in Davarzan County, in Razavi Khorasan Province, which showed an annual growth rate about three times higher than other clusters (Fig. 5).

### Space-time clusters

**Fig. 6:**
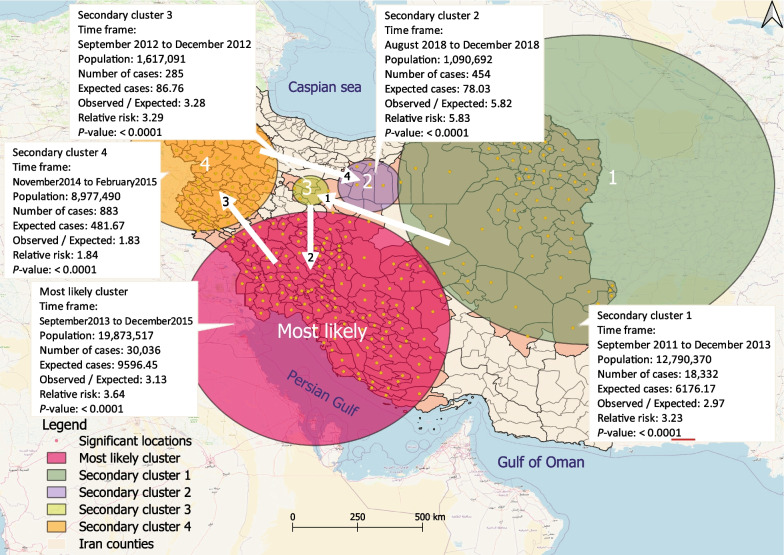
High-risk space–time clusters of cutaneous leishmaniasis cases in Iran between March 2011 and March 2020. The numerical value of the arrows denotes the spatiotemporal moving patterns of CL occurrence

Five significant space-time clusters (*P* < 0.05) of CL were identified in the study period. These clusters covered a total area of 1547 km, ranging in diameter from 60.8 to 638.5 km, and included approximately one-third of all patients (32.4%; 49,990) and 55.5% of the total population. No statistically significant spatiotemporal clusters were identified during the 2016–2017, 2017–2018, and 2019–2020 time periods. Figure 6 shows the cluster locations, and the cluster characteristics are described in the text comments. The most likely cluster, with a *LLR* of 15,347.5, encompassed 135 counties in the southern, south-western, and central-southern regions of Iran. This cluster showed a higher *LLR* from September 2013 to December 2015, indicating a higher incidence of CL in these regions during that period.

The geographic movement and spread of CL continued over the 9-year study period, affecting several regions of Iran. However, the clusters exhibited a distinct pattern of displacement in both space and time during the study period. The first cluster of cases occurred in the north-eastern and eastern regions of Iran between September 2011 and December 2013. In 2012, this disease also affected six counties in Qom and Markazi, two provinces in the central region of Iran. Subsequently, the geography of CL shifted from the center in the south-westerly direction, and clusters were detected in the southern, central-southern, southwestern, and western regions. Two years later, the epidemic shifted to Isfahan, Semnan, and Tehran in the central-northern region, and Cluster 2 had the highest *RR*.

## Discussion

This study aimed to reveal spatiotemporal movement patterns and extract spatially concealed incidence patterns of CL to identify high-risk zones. Identifying areas with high-risk rates is crucial for targeted epidemiological investigations, health policy interventions, prioritizing resource allocation, and to control CL occurrence [48, 49], particularly in countries with limited resources, such as Iran. Despite attempts to manage the disease, CL has expanded to previously unaffected regions of Iran over recent decades. Environmental factors could have played an important role in facilitating the spread of CL from endemic to non-endemic areas [50].

Our analysis indicates a decrease in CL incidence during the study period, consistent with the fixed seasonal pattern observed in Iran. Sand flies, the primary vector for the disease, are most active in the spring and summer [51, 52], resulting in a peak in CL incidence during autumn, particularly in October and November. This pattern has been consistently observed in prior investigations in Iran [26, 51, 53–55]. The incubation period for CL ranges from 2 weeks to 8 months [56], depending on the species, with the high occurrence of ulcers in autumn predictable due to the incubation period [57]. Therefore, it is recommended to wear full coverage in high-risk areas, particularly during spring and summer.

Purely spatial analysis revealed significant high-risk CL clusters in central, central-south, north-eastern, south-western, and western regions of Iran. Earlier studies indicate CL is dispersed in 25 of Iran’s 31 provinces, including the center, west, southwest, south, southeast, east, and northeast [14]. High frequencies of CL have also been found in southwest Iran [58]. CL frequency varies from 1.8 to 37.9% across provinces, with 62.6% of all cases in rural areas [23]. Detected clusters in this study included regions of Isfahan, Fars, Kerman, Yazd, Ilam, Khuzestan, Lorestan, Golestan, North Khorasan, Razavi Khorasan, Semnan, Damghan, and Kermanshah. Previous studies identified Ilam, Khuzestan, Fars, Khorasan Razavi, Yazd, Bushehr, and Isfahan as having the highest prevalence [24, 59].

The study revealed a spatial variation in temporal analysis, with an annual increase in the internal time trend of all clusters but a decline in the external time trend. This suggests an overall decrease in CL incidence, but with certain regions exhibiting an increasing tendency. The research identified clusters with an annual growth rate in several regions, including the north to north central, south, southeast, and west central areas of the country, which were not previously identified as statistically significant clusters by spatial analysis alone, despite the fact that the illness incidence in these regions is increasing and they can be expected as potential high-risk regions. The study projected a rising frequency of CL in several regions, including Tehran, Alborz, Mazandaran, Qom, Qazvin, Markazi, Hamedan, Chahar Mahal, Bakhtiari, Hormozgan, and Sistan Baluchestan. Factors such as agricultural activities and livestock breeding, large numbers of travellers attending religious ceremonies, effect of occupation, and climate variability were suggested as potential contributors to the increasing trend of CL in these regions [60–63].

The space–time analysis showed spatiotemporal high-risk clustering of CL, with clusters moving from northeast/east to central/southern/western regions and in a circular pattern, the concentration of cases shifted to the central-north regions. The latest active cluster included Tehran, Semnan, and Isfahan. Tehran, identified by spatial variation analysis as a location with a rising tendency, may become one of the most active CL hubs, in the near future. Tehran, the national capital, is one of the country’s most populous cities and plays a strategic role in the country’s economy and policy [64]. Immigration and unplanned urban growth, which are prevalent in Tehran, may contribute to the disease’s occurrence [24, 64, 65].

The prevalence of CL in Iran might be influenced by environmental, socioeconomic, and demographic factors. Poverty, inadequate housing, poor sanitary conditions, malnutrition, population mobility, occupational exposure, environmental changes, and climate change are significant risk factors for the disease, along with certain vocations such as mining, hunting, and agricultural activities [26, 55, 66–71]. Uncontrolled urbanization and development, urban migration, construction of new homes near historic structures, and widespread deforestation can also increase the risk of infection. These issues are causing severe health consequences and geographically heterogeneous CL distribution in Iran [24, 64, 65, 72–75].

CL incidence is higher in areas with daily rainfall, maximum/minimum temperature and humidity, and these environmental parameters are more prevalent in hyper-endemic regions than in low-endemic regions. The prevalence of CL was higher in the majority of North Khorasan and Razavi Khorasan, where precipitation, vegetation, specific humidity, evapotranspiration, and soil moisture were all higher than in the southern regions [69]. The large climate heterogeneity in areas like Khuzestan and Khorasan provinces might be the primary cause of the shift in CL epidemiology and transmission [67]. Urbanization, drought, and climate conditions also play a significant role in the spread of CL in high-risk regions such as Fars, Ilam, Kerman, Isfahan, and Yazd [24, 70, 72, 73, 75–79]. Urbanization damages the ecosystem of rodents and relocates them to residential areas. Planting Haloxylon and Atriplex, two plants well-adapted to arid and semi-arid areas and desert environments, to enhance soil quality, around the cities and villages of Kerman [76] and Yazd [77] causes the formation of rodent colonies in human residential areas. The accumulation of construction debris in urban areas attracts rodents as *Phlebotomus* reservoirs. As unintentional hosts of CL, stray canines living near residential areas and sand flies spread the disease [72]. In the rural areas, the majority of individuals are working and spending more time on farms and in animal husbandry, so they are susceptible to mosquito bites. These factors may play a significant effect in the high CL clustering in the provinces of Fars [24, 78], Semnan, Damghan, and Kermanshah [55, 71].

CL epidemics are linked to migration and contact with endemic areas, including Iran’s neighbors Afghanistan, Pakistan, and Iraq, as well as Syria and Saudi Arabia, where CL is also endemic [3, 65, 74, 80]. Iran has been a shelter for refugees for almost four decades [80]. About 780,000 registered and 2.3 million unregistered Afghans reside in Iran, with 96% residing in urban areas [65]. About 55% of Afghan immigrants reside in the provinces of Isfahan, Razavi Khorasan, and Tehran [65]. Moreover, 20,000 Iraqis have relocated to Iran [80]. Earlier investigations highlighted a significant CL outbreak in immigrant habitats [72, 74, 75], and our data indicate that CL has spread across the majority of Iranian counties.

Our study has important implications not only for Iran but also for other countries facing similar challenges with controlling the spread of CL. The methodology used in our study, which involved analyzing county-level incidence data using the SaTScan approach, is widely applicable and can be used in other settings to identify high-risk areas and develop targeted interventions to reduce disease transmission. In addition, the type of data we used, which is routinely collected in many countries, further enhances the generalizability of our findings. Future research might explore the potential applicability of our approach in other regions and settings to improve the understanding of CL epidemiology and support the development of evidence-based interventions.

This study has limitations in terms of the surveillance system, as it may underestimate the incidence of CL by not including patients with minor lesions who do not seek healthcare. The species of CL were not differentiated, and population data from 2016 was used to calculate rates due to lack of access to more recent data. However, the impact of using 2016 data is unlikely to be significant given its proximity to the middle of the study period. Also, Scan Statistics does have limitations that should be acknowledged. For instance, it is limited by its circular search window, which may not capture clusters that have irregular or non-circular shapes. Also, its sensitivity may be affected by the choice of the scan window size and the significance level used for cluster detection. Despite these limitations, Scan Statistics remains a valuable tool for detecting and characterizing spatial and temporal clusters of infectious diseases, and its use in public health research should be encouraged.

## Conclusions

This study provides valuable insights into the temporal, spatial, and spatiotemporal patterns of CL incidence, and identifies regions with distinct incidence trends, which can inform policymakers in designing interventions to prevent the spread of CL from endemic to non-endemic regions. The analysis revealed that CL cases exhibited significant circular spatiotemporal patterns, suggesting a complex aggregation movement that requires effective management. To better comprehend the spatial movement and changing patterns of CL incidence, further investigation is warranted to examine the contribution of sociodemographic, environmental factors, and sand fly reservoirs. Additionally, further research is necessary to examine the spatiotemporal trends of CL incidence and their correlation with environmental risk factors in provinces exhibiting an increasing incidence trend.

## Supplementary Information

**Additional file 1.** Map of the study area.

**Additional file 2.** Case file.

**Additional file 3.** Coordinate file.

**Additional file 4.** Population file.

**Additional file 5.** Spatial analysis layers.

## Data Availability

The cleaned data used for analysis are available to the public via Additional files 2, 3, 4, and 5.

## Abbreviations

CL: Cutaneous leishmaniasis
*RR*: Relative risk
*LLR*: Log likelihood ratio
*ITT*: Internal time trend
*ETT*: External time trend
