# Supplementary figures and images for: High-risk spatiotemporal patterns of cutaneous leishmaniasis: a nationwide study in Iran from 2011 to 2020
Source: Infect Dis Poverty. 2023 May 15;12:49. doi: 10.1186/s40249-023-01103-1 (PMC10184363; doi:10.1186/s40249-023-01103-1)

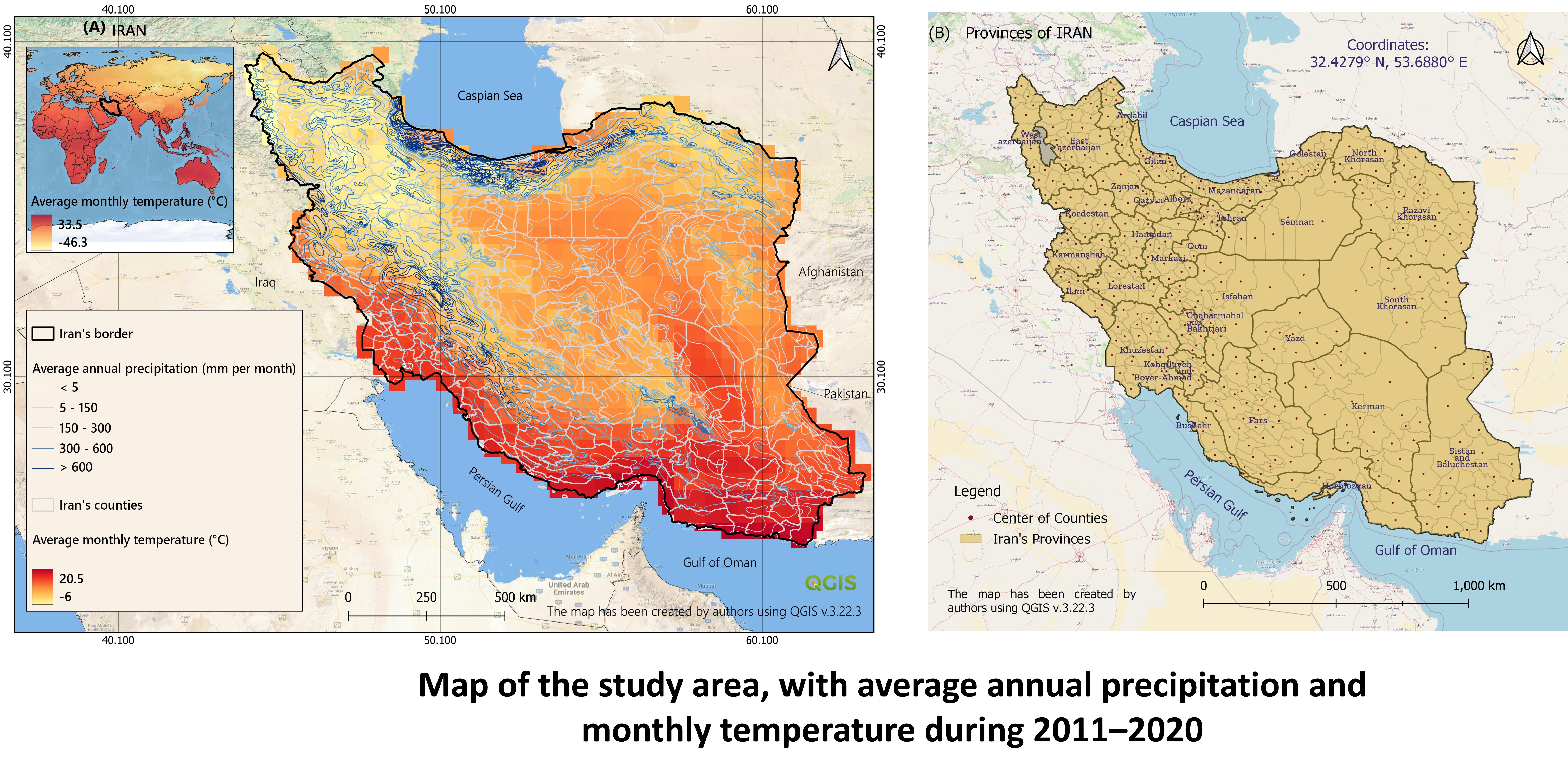

Supplement: Supplementary file 1 — Additional file 1. Map of the study area. [file 40249_2023_1103_MOESM1_ESM.jpg]
